# Analysis of the effect of meteorological factors on hemorrhagic fever with renal syndrome in Taizhou City, China, 2008–2020

**DOI:** 10.1186/s12889-022-13423-2

**Published:** 2022-06-01

**Authors:** Rong Zhang, Ning Zhang, Wanwan Sun, Haijiang Lin, Ying Liu, Tao Zhang, Mingyong Tao, Jimin Sun, Feng Ling, Zhen Wang

**Affiliations:** 1 Key Laboratory of Vaccine, Prevention and Control of Infectious Disease of Zhejiang Province, Zhejiang Provincial Center for Disease Control and Prevention, Zhejiang Province, Hangzhou, 310051 China; 2 Puyan Street Community Health Service Center of Binjiang District, Zhejiang Province, Hangzhou, 310013 China; 3 Taizhou City Center for Disease Control and Prevention, Zhejiang Province, Taizhou, 318000 China; 4 Ningbo University School of Medicine, Zhejiang Province, Ningbo, 315000 China

**Keywords:** Hemorrhagic fever with renal syndrome, Distributed lag non-linear, Generalized additive models, Lag effect, Interactive effect

## Abstract

**Background:**

Hemorrhagic fever with renal syndrome (HFRS) is endemic in Zhejiang Province, China, while few studies have concentrated on the influence of meteorological factors on HFRS incidence in the area.

**Methods:**

Data on HFRS and meteorological factors from January 1, 2008 to December 31, 2020 in Taizhou City, Zhejiang Province were collected. Multivariate analysis was conducted to the relationship between meteorological factors including minimum temperatures, relative humidity, and cumulative rainfall with HFRS.

**Results:**

The HFRS incidence peaked in November and December and it was negatively correlated with average and highest average temperatures. Compared with median of meteorological factors, the relative risks (RR) of weekly average temperature at 12 ℃, weekly highest temperature at 18 ℃relative humidity at 40%, and cumulative rainfall at 240 mm were most significant and RRs were 1.41 (95% CI: 1.09–1.82), 1.32 (95% CI: 1.05–1.66), 2.18 (95% CI: 1.16–4.07), and 1.91 (95% CI: 1.16–2.73), respectively. Average temperature, precipitation, relative humidity had interactions on HFRS and the risk of HFRS occurrence increased with the decrease of average temperature and the increase of precipitation.

**Conclusion:**

Our study results are indicative of the association of environmental factors with the HFRS incidence, probable recommendation could be use of environmental factors as early warning signals for initiating the control measure and response.

**Supplementary Information:**

The online version contains supplementary material available at 10.1186/s12889-022-13423-2.

## Background

Climate change, especially extreme weather, not only affect the incidence of acute infectious diseases of the respiratory tract [1–3], but also increase the risk of death in patients with chronic diseases [4]. Hemorrhagic fever with renal syndrome (HFRS) is a natural focal disease, and a large number of studies have shown that its incidence is influenced by climate change [5]. In the context of global warming, temperature, rainfall, and relative humidity are the main meteorological factors that pose a serious threat to human health [6]. Previous studies on the impact of meteorological factors on diseases have identified certain hysteresis effects, which vary in form and by region [7, 8].

Meteorological factors, such as temperature, precipitation, and humidity, might affect human travel, thereby directly affecting the likelihood of rodent-human contact [9]. They can also affect the spread of diseases by affecting crop yields, rodent reproduction, and vector density [10]. For example, temperature and rainfall were associated with the host ecosystem, affecting the HFTS transmission speed and the potential risk of outbreaks [11, 12]. These factors had a lagging effect on HFRS incidence, but lag time ranged 3–5 months in different areas [13–16]. Moreover, El Niño extreme weather events were also associated with the occurrence of HFRS [17].

The first documented case of HFRS in Zhejiang Province was reported in Jiaxing City in 1963. Since then, the size of the endemic area has gradually increased. In recent years, the number of cases has decreased with vaccination, rodent control strategies, and environmental sanitation improvements [18]. However, the affected areas of Zhejiang Province is gradually expanding, and the incidence rates in some areas remain high [19]. Up to date, cases have been reported in all 11 prefecture-level cities in the province [19]. However, few studies have concentrated on the influence of meteorological factors on HFRS incidence in the area. In this study, distributed lag nonlinearity (DLNM) and generalized additive model (GAM) were used to evaluate the impact of HFRS incidence in Taizhou City, Zhejiang Province, and to determine the key influencing factors.

## Material and methods

### Study area

Taizhou City, a coastal city in the central part of Zhejiang Province, belongs to the mid-subtropical monsoon area and experiences four distinct seasons (Supplementary Figure S1). The territory experiences mild summers, cold winters, abundant rain, and a mild, humid climate due to the meteorological effects of nearby ocean waters and mountains in the northwest.

### Data collection

According to the Law on Prevention and Treatment of Infectious Diseases, HFRS is classified as a Class B infectious disease in China, and cases must be reported within 24 h of diagnosis [19]. Data on HFRS from 2008 to 2020 in Taizhou City were collected from the Chinese Notifiable Disease Reporting System.

We collected daily meteorological data from the China Meteorological Data Sharing Service System (http://data.cma.cn/). These data, including daily average temperature, (Avetemp), minimum temperature (Mintemp), maximum temperature (Maxtemp), relative humidity, and total precipitation, were used to calculate the weekly average for each value.

### Statistical methods

Normality test and descriptive analysis were conducted to summarize characteristics of all variables. Spearman correlation was used to assess the relationship between HFRS incidence and meteorological factors. This study developed a time series model based on the GAM and used the cross-basis process to describe the distribution of changes in the independent variable dimension and the lag dimension simultaneously [8]. Further, DLNM was used to fit the non-linear and lag effects of weekly Avetemp, Maxtemp, Mintemp, average relative humidity, and cumulative rainfall on the risk of HFRS [20]. The incubation period for HFRS is affected by the host animal, vector density, and meteorological factors, and lasts for several weeks. In our study, the maximum lag period was set to 16 weeks [13, 20, 21]. Since HFRS cases in Taizhou City were relatively rare, Quasi-Poisson regression was used in this model to control for overdispersion. We used a two-stage analysis method. First, we used the DLNM to estimate the association of weekly Avetemp, Maxtemp, Mintemp, relative humidity, and weekly total precipitation (WTP) with the number of HFRS case [^22^]. The general algebraical definition of the model are as follows:

$$Log[E(Yt)] = \beta + cb(Kt,16,\beta 1) + S1(x) + S2(z)+S3(m)+S4(n)+ S5(week)$$

Among them, t is the observation week; [*E(Yt)*] is HFRS cases observed in month Yt, βis the intercept of the entire model; cb (Kt, 16, and β1) is the cross-basis function of K, and K is one of the meteorological elements. S1–4 are factors such as Avtemp, Maxtemp, Mintemp, RH, and WTP; β1 is the estimated value of the effect of K in a specific lag week t; the maximum lag week is set to 16; Week is the ordinal variable of the week in the year; s() is the penalty spline function. This study uses cubic spline functions, s1–4, to adjust the confounding factors in the model, and s5(week) to adjust the weekly confounding factors.

Second, we analyzed the interaction between weekly Avetemp, Maxtemp, Mintemp, relative humidity, and accumulated rainfall with GAMs, and then analyzed the different effects of high and low values of the meteorological factors on the cases. The basic model as follows:

$$Log[\mathrm{E}(\mathrm{Yt})] =\upbeta 2 +\mathrm{ S}1(\mathrm{k},\mathrm{x}) +\mathrm{ S}2(\mathrm{z}) +\mathrm{ S}3(\mathrm{m}) +\mathrm{ S}4(\mathrm{n}) +\mathrm{ S}5(\mathrm{week})$$

β2 is the intercept; K is one of the meteorological factors (Avtemp, Maxtemp, Mintemp, RH, and WTP), and X, Z, M, and N denote the other factors. s() means the penalty spline function. s1 (K and X) is the spline function of the interaction between variables K and X.

In the model, the number of cases was used as the dependent variable, and a cross-basis function was established for the number of cases and temperature. Spline interpolation was used to control the influence of confounding factors such as relative humidity, rainfall, and long-term trends. The best degree of freedom (df) was selected based on the spline function results through sensitivity testing and generalized cross-validation criteria [23].

DLNM can describe the complex nonlinearity and hysteresis correlation of temperature-HFRS through the cross basis function. It is necessary to scientifically define the reasonable lag time of the model [^21^]. We chose one ns (natural cubic B-spline, df = 6) as the exposure–response. Two nodes are located at the 2.5^th^ and 97.5^th^ percentiles of the meteorological factor distribution, and the other is for the exposure–response relationship, based on high temperature [13, 15]. The assumption that Maxtemp and Mintemp may affect the incidence of HFRS is fixed (df = 6) and the maximum lag time was set at 16 weeks to capture the delayed effects of extreme temperatures. In this study, the degrees of freedom and maximum lag times for mean temperature, relative humidity, accumulated rainfall, mean maximum temperature and mean minimum temperature were set in order: (df = 6, lag = 16; df = 4, lag = 16; df = 3, lag = 10; df = 6, lag = 21; df = 4, lag = 20).

We performed sensitivity analysis by changing the df of the weather variables and time points. All analyses were performed using ArcGIS 10.2 (ESRI, Redlands, CA, USA) and R software (packages "dlnm" and "mgcv") (R Foundation for Statistical Computing, Vienna, Austria).

## Results

**Fig. 1:**
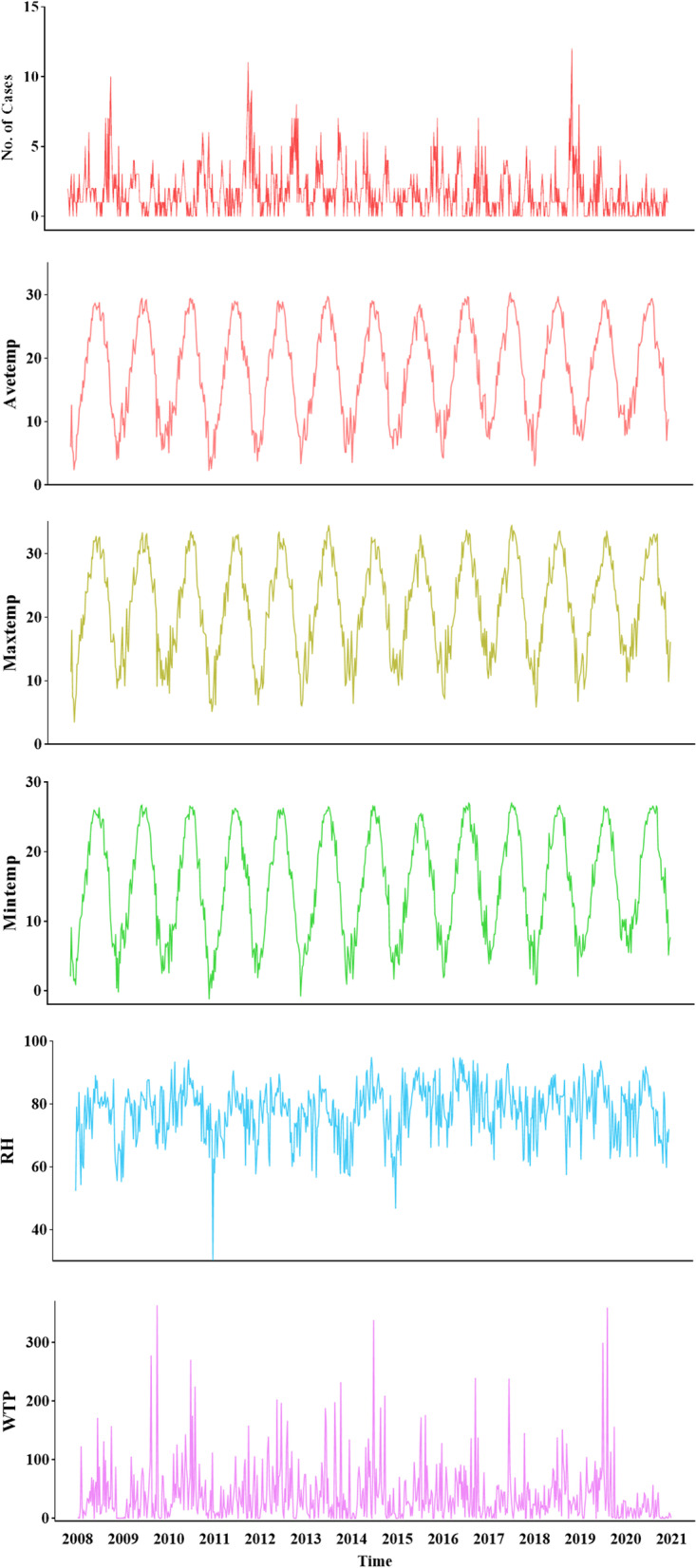
Time series of weekly Avetemp,Tmax, Tmin, RH and WTP, and number of HFRS from 2008 to 2020 in Taizhou City, China

**Fig. 2:**
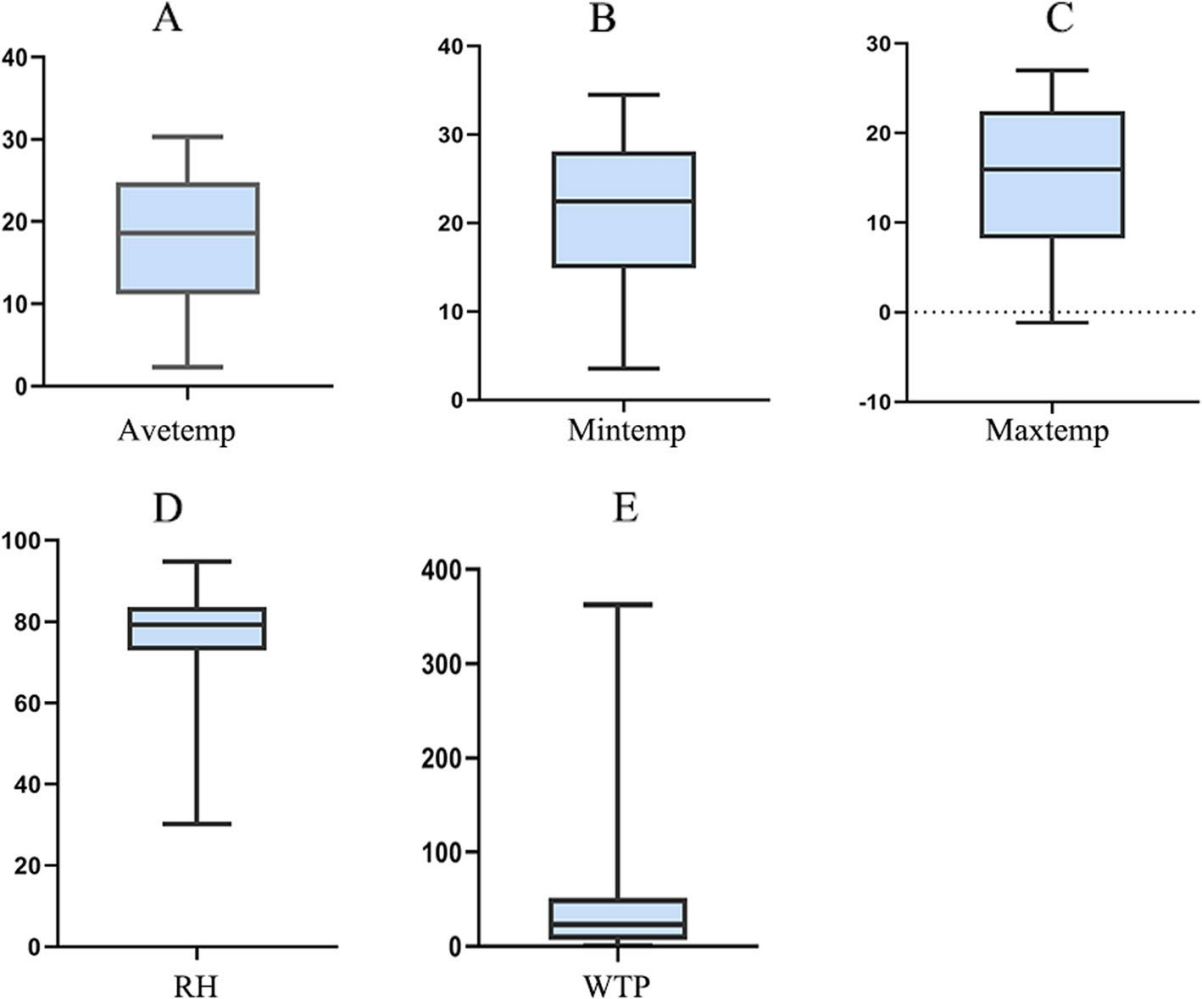
Boxplot of Avetemp, Mintemp, Max temp, RH and WTP

During the study period, a total of 1196 HFRS cases were reported in Taizhou City. Descriptive statistics collected over the past 13 years indicated that the highest weekly case distribution in Taizhou reached 12 cases (Table 1). The Avetemp, Maxtemp, and Mintemp in Taizhou City from 2008 to 2020 all show a normal distribution and showed obvious periodicity and seasonality (Fig. 1). The average weekly temperature was 18.04 °C; Mintemp, 2.33 °C, and Maxtemp, 30.33 °C. The weekly average Maxtemp and weekly average Mintemp were 19.3 °C and 11.13 °C, respectively. The average weekly humidity was 77.88% and the average weekly rainfall was 38.54 mm (Fig. 2). HFRS incidence was negatively correlated with the Avetemp and the highest Avetemp, while it didn’t significantly related to the weekly average relative humidity, weekly total precipitation, and the lowest Avetemp (Table 2).

**Table 1:** Descriptive statistics of weekly HFRS cases and meteorological factors in Taizhou City, China from 2008 to 2020

Variable	*X* ± S.D	Min	P2.5	P25	P50	P75	P97.5	Max
Cases	1.74 ± 1.8	0.00	0.00	0.00	1.00	2.00	7.00	12.00
Avetemp(℃)	18.04 ± 7.61	2.33	4.55	11.18	18.63	24.78	29.14	30.33
RH(%)	77.88 ± 8.57	30.25	58.56	72.96	79.18	83.71	91.61	94.75
WTP(mm)	38.54 ± 48.85	0.00	0.00	6.79	23.11	51.57	173.60	362.64
Maxtemp(℃)	19.3 ± 7.65	3.54	14.91	14.91	22.47	28.06	33.20	34.48
Mintemp(℃)	11.125 ± 7.73	-1.18	1.91	8.25	15.95	22.40	26.42	27.01

*Abbreviations*: *S.D* The standard deviation, *Min* The minimum of variables, *Max* The maximum of variables, *Avetemp* Average weekly temperature, *RH* Weekly average relative humidity, *WTP* Weekly total precipitation, *Maxtemp* Maximum average weekly temperature, *Mintemp* Minimum average weekly temperature

**Table 2:** Correlation analysis of meteorological factors and HFRS in Taizhou city,China from 2008 to 2020

Variable	Cases	Avetemp	RH	WTP	Tempmax	Tempmin
Cases	1	-0.08 *	-0.03	0.02	-0.08*	-0.07
Avetemp		1	0.40 *	0.20*	0.96 *	0.96*
RH			1	0.64*	0.32*	0.36*
WTP				1	0.19*	0.22*
Tempmax					1	0.98*
Tempmin						1

*Abbreviations*: *Avetemp* Average temperature, *CI* Confidence interval, *df* Degree of freedom, *DLNM* Distributed lag non-linear model, *GAM* Generalized additive model, *HFRS* Hemorrhagic fever with renal syndrome, *Maxtemp* Maximum temperature, *Mintemp* Minimum temperature, *RH* Relative humidity, *WTP* Weekly total precipitation, *RR* Relative risk

^*^p < 0.05

**Fig. 3:**
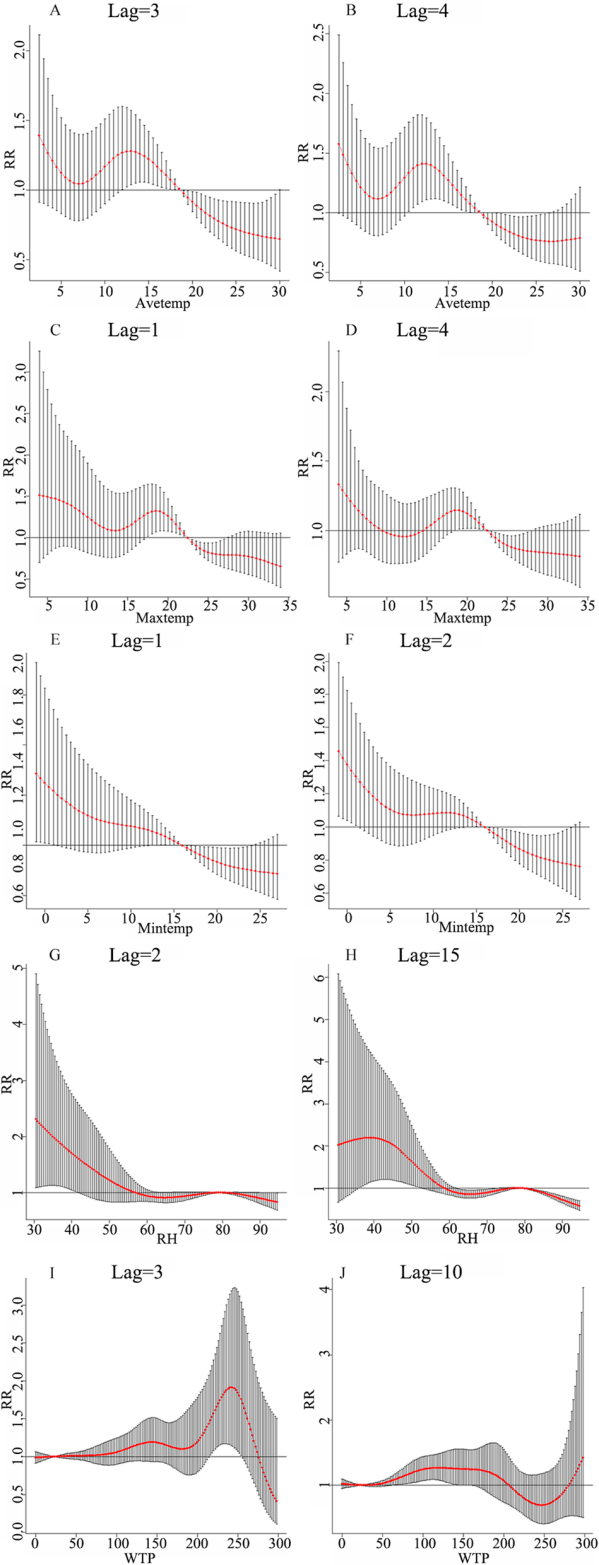
The lag-specific effect of meteorological factors on HFRS in Taizhou City

In the DLNM, we used the median of each meteorological factor as a reference and then calculated the relative risk of each variable. The impact of Avetemp on HFRS rapidly decreased and then slowly increased. In Lag 3, weekly Avetemp is most significant at 13 °C (RR = 1.28, 95% CI = 1.04–1.57). In Lag 4, weekly Avetemp was most significant at 12 °C (RR = 1.41, 95% CI = 1.09–1.82) (Fig. 3A, B). In lag 3, Maxtemp is most significant at 18 °C (RR = 1.32, 95% CI = 1.05–1.66) (Fig. 3C). In lag 4, Maxtemp is most significant at 20 °C (RR = 1.12, 95% CI = 1.02–1.24) (Fig. 3D). There was no statistical difference between the high and low values of the Mintemp; the Mintemp of 1 °C had the same RR value in lag1 and lag2 (RR = 1.59, 95% CI = 1.02–2.47) (Fig. 3E, F).

The RR of the highest value of relative humidity in the 97.5^th^ percentile in lag2 and lg16 were 0.97, and 0.61, respectively (Supplementary Fig. S2G, H). A relative humidity of 31% was the most significant in lag 2 and a maximum relative humidity of 40% was the most significant in lag 15 (Fig. 3G, H).

The highest WTP value of the 97.5^th^ percentile had the largest RR in lag13 (RR = 1.35, 95% CI = 1.01–1.79) (Supplementary Fig. S2I, J); a cumulative rainfall of 240 mm in lag3 was the most significant (RR = 1.91, 95% CI = 1.16–2.73) (Fig. 3I). A cumulative rainfall of 120 mm in lag10 was the most significant (RR = 1.27, 95% CI = 1.08–1.49) (Fig. 3J).

**Fig. 4:**
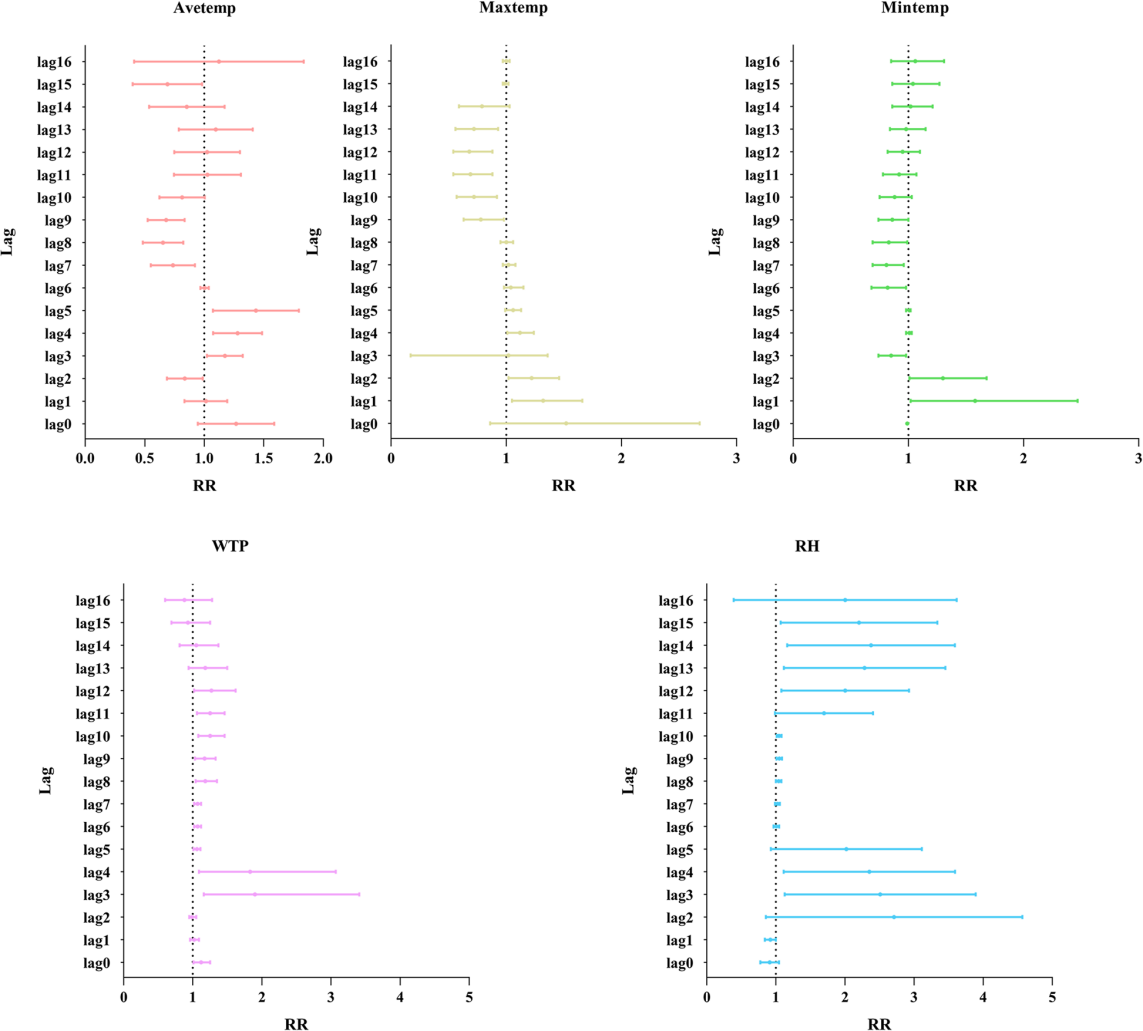
RRs of meteorological factors on HFRS at different lags in Taizhou city from 2008 to 2020

We calculated the corresponding RR for the minimum to the maximum lag of each meteorological factor (Fig. 4), and the Avetemp was between lag3–5 (RR = 1.4, 95% CI = 1.09–1.81). The time effect of the Maxtemp was significant, and the risk of infection was the highest in the lag1–2 weeks. Regarding cumulative rainfall, the effect of lag was most significant during lag3–4 weeks. The maximum effect of relative humidity was the most significant at lag3–4 and lag12–15 weeks, respectively.

**Fig. 5:**
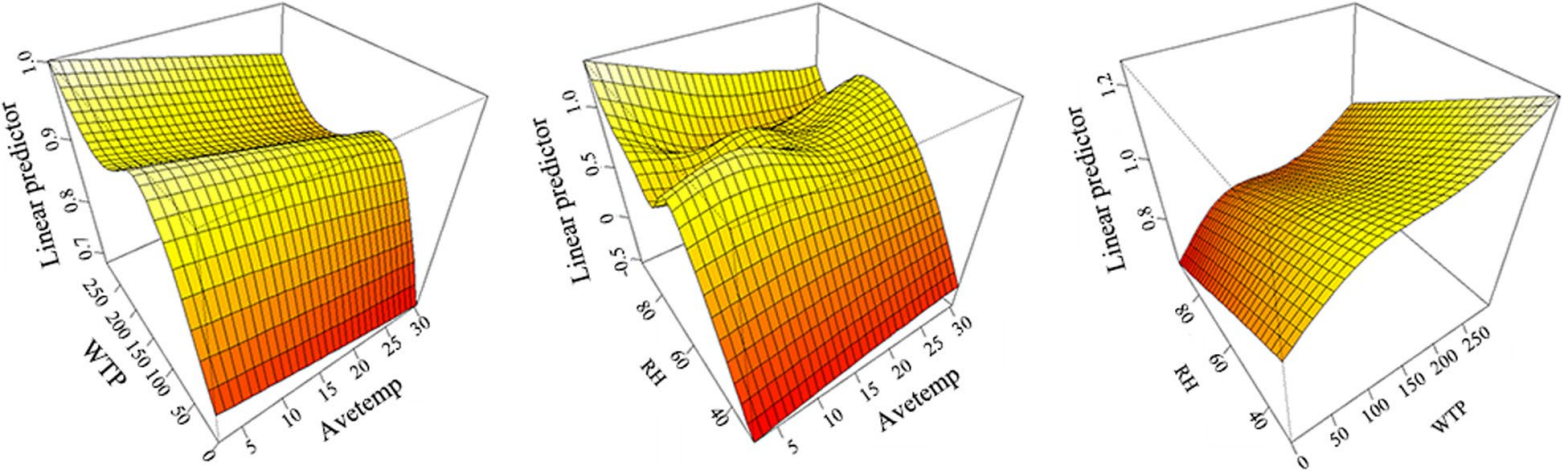
The coefficients of meteorological factors on HFRS in Taizhou City

GAMs was used to explore the interaction between Avetemp, WTP, and RH, and the results are shown in Fig. 5. The left side of Fig. 5 showed the interaction between Avetemp and WTP. Obviously, the infection risk of HFRS was inversely proportional to Avetemp and directly proportional to the WTP. As showed in the middle of Fig. 5, the infection risk of HFRS is inversely proportional to Avetemp and directly proportional to RH. Figure 5 showed that as the WTP increased, the RH decreased and the risk of infection increased. The risk of HFRS infection increased with the decrease of Avetemp and the increase of WTP. These indicated that HFRS in Taizhou City increased when Avetemp decreased and WTP increased.

## Discussion

In this study, we investigated the relationship between Avetemp, Maxtemp, Mintemp, WTP, relative humidity and HFRS in Taizhou City from 2008 to 2020 using DLNM and GAMs. Our study found that weekly Avetemp and weekly maximum temperature were negatively associated with HFRS incidence, which is consistent with results from Shandong Province [24].

The lagged effects of WTP and relative humidity were also most pronounced in Taizhou City, with a lag of 3–4 weeks. Rather than concentrating rodent control efforts only twice, in winter and spring [7, 19], the high incidence period identified in this study. Several studies have confirmed that extreme weather has a significant impact on many diseases [18, 19]. We found that the effects of Maxtemp and Mintemp on HFRS were most pronounced at a lag of 1 week. Several models have been used to study the effect of later factors on dengue fever, and similarly confirmed the existence of a lag period for climatic factors on local dengue incidence [25].

We found that the risk of infection increased with the increase of precipitation, which was similar with previous findings [26]. The effect of WTP on the risk of disease in Taizhou City was most pronounced at a lag of 1 month, and this effect persisted until a lag of 12 weeks. This study confirmed that infectious diseases in coastal areas such as Zhejiang Province were more affected by tropical cyclones [27]. For example, rainfall and relative humidity had a significant effect on severe fever with thrombocytopenia syndrome [28].

## Conclusion

Meteorological factors had non-line relationship with HFRS and lag effects exist. HFRS mostly occurred when temperature and relative humidity were low and WTP was high. Our study results are indicative of the association of environmental factors with the HFRS incidence, probable recommendation could be use of environmental factors as early warning signals for initiating the control measure and response.

### Limitations

HFRS incidence was directly associated with density and infection rate of rodents, but these data were not available in this study. More over, other factors including social factors and environmental factors might also influence HFRS. Further research should be conducted to explore the contribution rate of different factors on HFRS.

## Supplementary Information

**Additional file 1: Figure S1. **Study areas of Taizhou City in China. The map was created by ArcGIS 10.2 (Software, ESRI Inc., Redlands, CA, USA). The base layer of the map of Zhejiang Province was supported from National Earth System Science Data Center, National Science & Technology Infrastructure of China (http://www.geodata.cn). **Figure S2.** With the median as the reference, the lag effect between Avetemp, Maxtemp, Mintemp, RH and WTP and HFRS infection. Abbreviations: Avetemp, average temperature; CI, confidence interval; df, degree of freedom; DLNM, distributed lag non-linear model; GAM, generalized additive model; HFRS, Hemorrhagic fever with renal syndrome; Maxtemp, maximum temperature; Mintemp, minimum temperature; RH,relative humidity;WTP,weekly total precipitation; RR, relative risk.

## Data Availability

All data analyzed during this research period are included in the body of this article and supplementary materials.
