# Supplementary material for: Analysis of the effect of meteorological factors on hemorrhagic fever with renal syndrome in Taizhou City, China, 2008–2020
Source: BMC Public Health. 2022 Jun 1;22:1097. doi: 10.1186/s12889-022-13423-2 (PMC9161505; doi:10.1186/s12889-022-13423-2)
Supplement: Supplementary file 1 — Additional file 1: Figure S1. Study areas of Taizhou City in China. The map was created by ArcGIS 10.2 (Software, ESRI Inc., Redlands, CA, USA). The base layer of the map of Zhejiang Province was supported from National Earth System Science Data Center, National Science & Technology Infrastructure of China (http://www.geodata.cn). Figure S2. With the median as the reference, the lag effect between Avetemp, Maxtemp, Mintemp, RH and WTP and HFRS infection. Abbreviations: Avetemp, average temperature; CI, confidence interval; df, degree of freedom; DLNM, distributed lag non-linear model; GAM, generalized additive model; HFRS, Hemorrhagic fever with renal syndrome; Maxtemp, maximum temperature; Mintemp, minimum temperature; RH,relative humidity;WTP,weekly total precipitation; RR, relative risk. [file 12889_2022_13423_MOESM1_ESM.doc]

**Supplementary material**

FigureS1-3

**FigureS1.**Study areas of Taizhou City in China. The map was created by ArcGIS 10.2 (Software, ESRI Inc., Redlands, CA, USA).

The base layer of the map of Zhejiang Province was supported from National Earth System Science Data Center, National Science & Technology Infrastructure of China (http://www.geodata.cn).


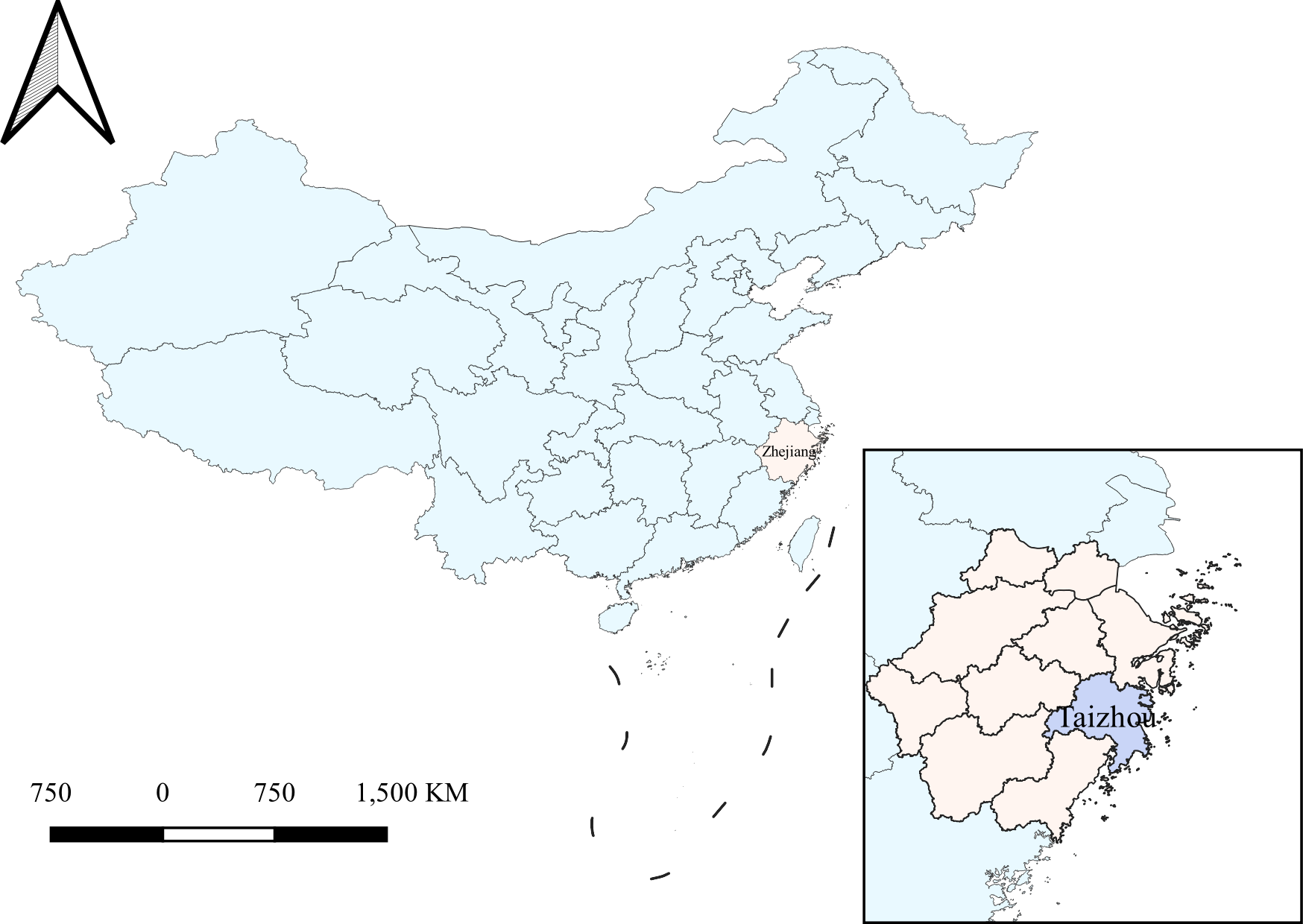


**FigureS2.**With the median as the reference, the lag effect between Avetemp, Maxtemp, Mintemp, RH and WTP and HFRS infection


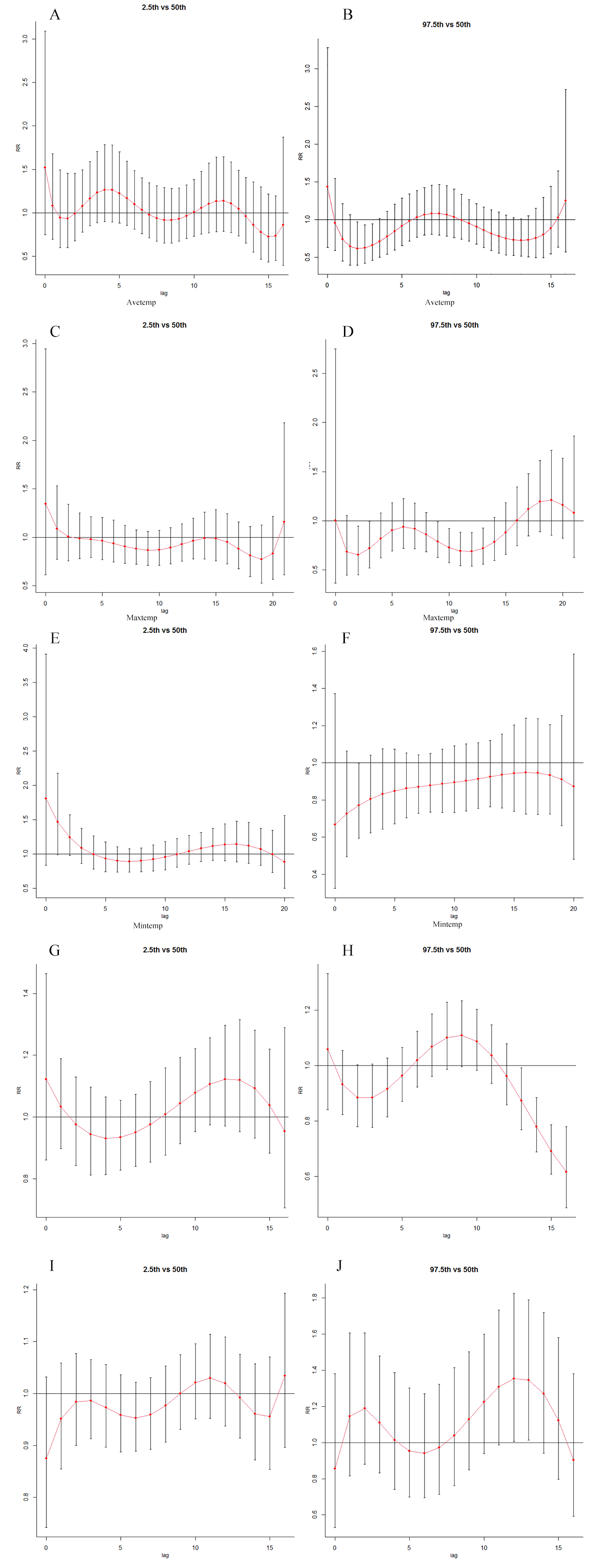


**FigureS3**.The cross correlation coefficients between climatic variables and HFRS cases in Taizhou city


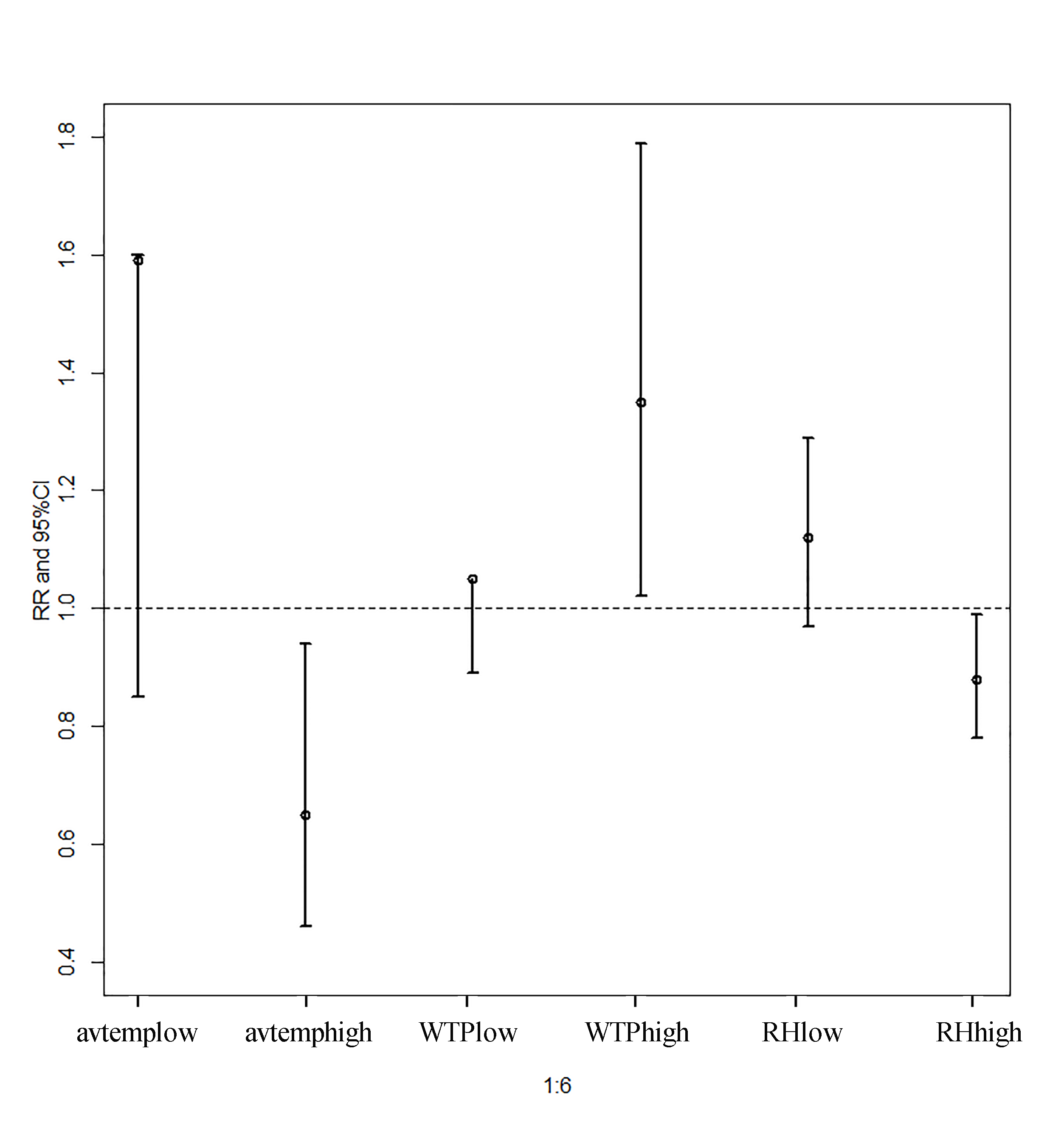


| **Table S1**. Descriptive statistics of weekly HFRS cases and meteorological factors in Taizhou City, China from 2008 to 2020. | | | | | | | | |
| --- | --- | --- | --- | --- | --- | --- | --- | --- |
| Variable | *`X*±S.D. | Min | P2.5 | P25 | P50 | P75 | P97.5 | Max |
| Cases | 1.74±1.8 | 0.00 | 0.00 | 0.00 | 1.00 | 2.00 | 7.00 | 12.00 |
| Avetemp（℃） | 18.04±7.61 | 2.33 | 4.55 | 11.18 | 18.63 | 24.78 | 29.14 | 30.33 |
| RH（%) | 77.88±8.57 | 30.25 | 58.56 | 72.96 | 79.18 | 83.71 | 91.61 | 94.75 |
| WTP（mm) | 38.54±48.85 | 0.00 | 0.00 | 6.79 | 23.11 | 51.57 | 173.60 | 362.64 |
| Maxtemp（℃） | 19.3±7.65 | 3.54 | 14.91 | 14.91 | 22.47 | 28.06 | 33.20 | 34.48 |
| Mintemp（℃） | 11.125±7.73 | -1.18 | 1.91 | 8.25 | 15.95 | 22.40 | 26.42 | 27.01 |

Abbreviations: S.D., the standard deviation; Min, the minimum of variables; Max, the maximum of variables；Avetemp,average weekly temperature;RH,weekly average relative humidity;WTP,weekly total precipitation;Maxtemp,maximum average weekly temperature;Mintemp,minimum average weekly temperature

Table S2.Correlation analysis of meteorological factors and HFRS in Taizhou city ,China from 2008 to 2020

| **Variable** | Cases | Avetemp | RH | WTP | Tempmax | Tempmin |
| --- | --- | --- | --- | --- | --- | --- |
| Cases | 1 | -0.08 * | -0.03 | 0.02 | -0.08* | -0.07 |
| Avetemp |  | 1 | 0.40 * | 0.20* | 0.96 * | 0.96* |
| RH |  |  | 1 | 0.64* | 0.32* | 0.36* |
| WTP |  |  |  | 1 | 0.19* | 0.22* |
| Tempmax |  |  |  |  | 1 | 0.98* |
| Tempmin |  |  |  |  |  | 1 |

**Abbreviations**: Avetemp, average temperature; CI, confidence interval; df, degree of freedom; DLNM, distributed lag non-linear model; GAM, generalized additive model; HFRS, Hemorrhagic fever with renal syndrome; Maxtemp, maximum temperature; Mintemp, minimum temperature; RH,relative humidity;WTP,weekly total precipitation;RR, relative risk.
